# Reliability Analysis of FRP-Confined Concrete at Ultimate using Conjugate Search Direction Method

**DOI:** 10.3390/polym12030707

**Published:** 2020-03-23

**Authors:** Behrooz Keshtegar, Aliakbar Gholampour, Togay Ozbakkaloglu, Shun-Peng Zhu, Nguyen-Thoi Trung

**Affiliations:** 1 Division of Computational Mathematics and Engineering, Institute for Computational Science, Ton Duc Thang University, Ho Chi Minh City 800010, Vietnam; nguyenthoitrung@tdtu.edu.vn; 2 Faculty of Civil Engineering, Ton Duc Thang University, Ho Chi Minh City 800010, Vietnam; 3 Department of Infrastructure Engineering, The University of Melbourne, Victoria 3010, Australia; agholampour@unimelb.edu.au; 4 Ingram School of Engineering, Texas State University, San Marcos, TX 78666, USA; 5 School of Mechanical and Electrical Engineering, University of Electronic Science and Technology of China, Chengdu 611731, China; zspeng2007@uestc.edu.cn

**Keywords:** fiber-reinforced polymer (FRP), safety level, reliability analysis, model error, FRP-confined concrete

## Abstract

In this paper compressive strength and ultimate strain results in the current database of fiber-reinforced polymer (FRP)-confined concrete are used to determine the reliability of their design space. The Lognormal, Normal, Frechet, Gumbel, and Weibull distributions are selected to evaluate the probabilistic characteristics of six FRP material categories. Following this, safety levels of the database are determined based on a probabilistic model. An iterative reliability method is developed with conjugate search direction for evaluating the reliability. The results show that Lognormal and Gumbel distributions provide best probability distribution for model errors of strength and strain enhancement ratios. The developed conjugate reliability method provides improved robustness over the existing reliability methods owing to its faster convergence to stable results. The results reveal that the part of the database containing normal strength concrete (NSC) heavily confined (i.e., actual confinement ratio (*f_lu,a_*/*f’_co_*) > 0.5) by low and normal modulus carbon fibers (i.e., fiber elastic modulus (*E_f_*) ≤ 260 GPa) and moderately confined (i.e., 0.3 ≤ *f_lu,a_*/*f’_co_* ≤ 0.5) by aramid fibers exhibits a very high safety level. The segments of the database with a low and moderate safety level have been identified as i) NSC moderately and heavily confined by higher modulus glass fibers (i.e., *E_f_* > 60 GPa), ii) high strength concrete (HSC) moderately and heavily confined (i.e., *f_lu,a_*/*f’_co_* > 0.3) by glass fibers, iii) HSC lightly confined (i.e., *f_lu,a_*/*f’_co_* ≤ 0.2) by carbon fibers, and iv) HSC lightly confined by aramid fibers. Additional experimental studies are required on these segments of the database before they can be used reliably for design and modeling purposes.

## 1. Introduction

Using fiber-reinforced polymer (FRP) in enhancing seismic performance of concrete members has been extensively studied [1,2]. Lateral confinement of concrete using FRP wrap or tube causes an improvement in the ductility and strength of concrete [3,4,5]. Numerous studies were performed to investigate the compressive strength (*f′_cc_*) and ultimate axial strain (*ε_cu_*) of FRP-confined concrete (e.g., [6,7,8,9,10,11,12]). Different confinement models were developed to predict *f′_cc_* and *ε_cu_* (as the ultimate condition) using experimental datasets for design guidelines and practical applications [13,14,15,16,17,18]. In these models, the properties of FRP material, such as fiber type and thickness, have been considered as the most important parameters in the prediction of *f′_cc_* and *ε_cu_* of concrete confined by FRP layers [9]. Nevertheless, the accuracy and robustness of them depended on the size and reliability of the database of test results they were developed from.

Reliability analysis is an essential tool for ensuring robust design and evaluating safe condition of a structural element [19]. There are several probabilistic methods to evaluate the uncertainty using the reliability analysis theory, e.g., the analytical approaches using first-order/second-order reliability method (FORM/SORM) [20,21,22,23,24,25,26,27], Monte Carlo simulation (MCS) [28], weighted average simulation [29], and subset simulation [30]. Although the MCS method is commonly used to evaluate the reliability index owing to its accuracy and simplicity, this approach needs a large number of data points to obtain an accurate reliability index of a very small failure domain [31]. On the other hand, the FORM is extensively utilized in the engineering reliability analysis because of the good balance it offers between accuracy and time efficiency [21,26]. The other simulation methods have the complex formulation to approximate the failure domain and need extra details for evaluating the safe/failure domain [32].

Several iterative schemes have been used to estimate the reliability index of different problems based on the FORM. Val [33] used the original FORM formula to study the reliability of FRP-confined reinforced concrete columns and found the reliability of these columns highly dependent on the confinement level. Sadeghian et al. [34] also used the original FORM for analysis of reliability of FRP-confined reinforced concrete beams under flexural loading. Rasheed et al. [35] conducted the reliability analysis of girders of China-Pakistan Economic Corridor bridge using the original FORM formula. Gong and Yi [22] used Hasofer-Lind and Rackwitz-Fiessler (HL-RF) [36] and finite-step length (FSL) methods to determine the reliability index of shear strength of bridge structures and found that HL-RF converges faster than FSL. Liu and Der Kiureghian [37] introduced a more robust version of HL-RF as improved HL-RF (IHL-RF). Yang [21] used the stability transformation method (STM) with chaos control (CC) and reported that this method controlled the chaos and periodic oscillation of the FORM iterative algorithm. Keshtegar [26] and Keshtegar and Miri [38] used conjugate HL-RF (CHL-RF) for analyzing corroded pipes and conical structure and found that CHL-RF improved the convergence speed of FORM for nonlinear problems. Baji et al. [39] conducted reliability analysis of reinforced concrete columns retrofitted with FRP wrap under eccentric axial loading. They applied the advanced first-order Second moment method for reliability analysis. Zhang et al. [40] used HL-RF method for reliability analysis of FRP-to-concrete bonded joints. The efficiency of these methods in achieving stable results from an iterative algorithm makes the FORM reliable in the estimation of the failure probability. IHL-RF, CC, and FSL algorithms showed a more robust performance compared to HL-RF, however, they provided inefficient computational burden for highly nonlinear problems [26]. The CHL-RF method [38] is an efficient and robust method providing stable results for highly nonlinear problems [26], but it provides inefficient computational burden for highly nonlinear problems. These improved FORM versions based on steepest descent search direction were formulated for enhancing the accuracy of the original FORM. However, the efficiency of FORM is dependent on the parameters used in the reliability analysis.

For predicting the properties of FRP-confined concrete at ultimate accurately, it is important to use a large test database. However, using a large test database in not enough for accuracy of a proposed model and it is also necessary to establish the reliability of such a database using an efficient reliability method [41]. Recently, the conjugate search direction with adaptive formulation was developed for enhancement of the accuracy and efficiency of the FORM [42,43,44]. The literature review has shown that no study has been conducted to date on the application of a conjugate search direction method based on FORM (CFORM) on the reliability analysis of test databases of FRP-confined concrete.

In this study, a CFORM is developed with more robust and efficient performance, in terms of accuracy and computational time, in comparison to the existing methods to determine the reliability index of the current database of FRP-confined concrete and identify its segments that require improvements. Four safety levels are suggested based on the reliability analysis of the data as the reliability levels of the database to provide guidance in the design and modelling of FRP-confined concrete.

## 2. Research Significance

The focus of this study is (i) developing a new conjugate reliability method with a higher robustness over the existing reliability methods for highly nonlinear problems and (ii) using the developed method to determine the safety level of the current database of *f′_cc_* and *ε_cu_* of FRP-confined concrete columns. Results of this research make a significant contribution towards determining the reliability of the database, and establishing which parts of the database require additional experimental studies before they can be used reliably for design and modeling of FRP-confined concretes.

## 3. Reliability Analysis-Based Conjugate Search Direction

The main aim of FORM is finding the most probable point (MPP) as the nearest point on the limit state surface to the origin in the normal standard space. HL-RF as the traditional iterative FORM produces unstable and chaotic results [21,25,26,45]. On the other hand, the modified version of FORM requires a line search rule to compute a step size based on the Armijo [38,46], Wolfe condition [25], or merit function [37]. Thus, these improved FORM versions are more robust compared to HL-RF, however, they require additional iterations for evaluating a suitable step size, especially for highly nonlinear problems. FSL [22] and CC [21,45] methods are computationally inefficient as they require a smaller step size than that in HL-RF (i.e., less than 1) for stable results. CHL-RF algorithm is based on a conjugate search direction using Fletcher and Reeves (FR) conjugate scalar factor, providing stable results for highly nonlinear problems more inefficiently than FSL and HL-RF [47]. The CHL-RF is improved based on a limited FR (LFR) and dynamical chaotic finite-step size in chaotic conjugate control (CCC) to enhance its efficiency. However, the basic formulation of the conjugate search direction is extended by the FR scalar factor and therefore the effects of the previous and new grained vectors are not considered in computing the conjugate search direction [47]. An iterative FORM algorithm based on conjugate search direction is developed in this section for the improvement of the efficiency and robustness of FORM without utilizing line search rules. This algorithm is validated by three nonlinear limit state functions and the converged results from the proposed CFORM are compared with those of HL-RF, CC, FSL, directional stability transformation method (DSTM), CHL-RF, CCC, and LFR to illustrate its performance.

### 3.1. Conjugate Iterative Formula of FORM

**Figure 1:**
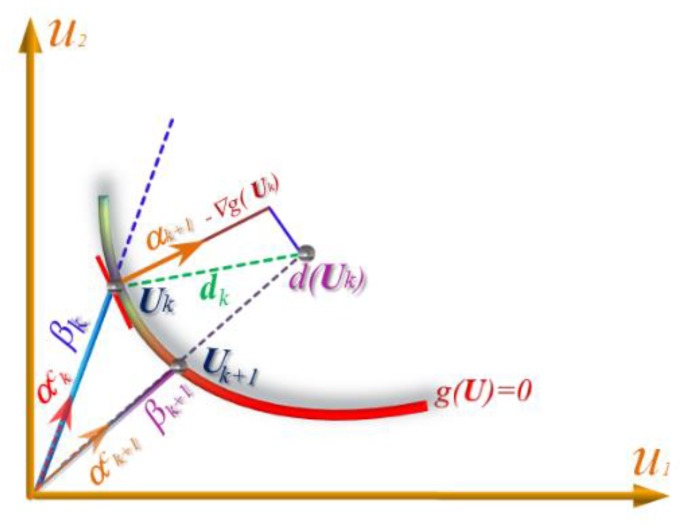
Schematic illustration of the iterative process of the CFORM algorithm.

The failure probability (*P_f_*) in the reliability analysis is estimated by Equation (1) [25].

(1) $$P_{f}=\int_{g\left(X\right)\leq 0}\ldots \int f_{X}\left(x_{1},\ldots ,x_{n}\right)dx_{1}\ldots dx_{n}=\Phi \left(-\beta \right),$$

in which *g*(*X*) is the limit state function, separating the domain of design into failure (*g*(*X*) < 0) and safe (*g*(*X*) > 0) regions with respect to various uncertainties using the random variables of *X = (x_1_,x_2_,…,x_n_*)*^T^*. *f_X_* is the joint probability density function of random variables, Φ is the standard normal cumulative distribution function, and *β* is the reliability index. The iterative CFORM formula to search MPP is given as follows [26,38]:

(2) $$U_{k+1}=\frac{\nabla^{T}g\left(U_{k}\right)U_{k}-g\left(U_{k}\right)}{\nabla^{T}g\left(U_{k}\right)\alpha_{k+1}^{C}}\alpha_{k+1}^{C}$$

in which U is the vector of normal standard random variables and $\alpha_{k+1}^{C}$ is the vector of conjugate unit normal at design point of U*_k_*. For reducing the parallel risk of the unit normal vector (α*_k_*_+1_) with the search direction vector, α*_k_*_+1_ is proposed based on the conjugate search direction by $\alpha_{k+1}^{C}$, which is computed as:

(3) $$\alpha_{k+1}^{C}=\frac{d\left(U_{k}\right)}{\left||d\left(U_{k}\right)|\right|}$$

where *d*(U*_k_*) states the point-based conjugate search direction and is defined by Equation (4).

(4) $$d\left(U_{k}\right)=U_{k}+d_{k}$$

where *d_k_* is the vector of conjugate search direction defined as:

(5) $$d_{k}=-\nabla g\left(U_{k}\right)+\frac{{\left||\nabla g\left(U_{k}\right)|\right|}^{2}-0.1\nabla^{T}g\left(U_{k}\right)\nabla g\left(U_{k-1}\right)}{{\left||\nabla g\left(U_{k-1}\right)|\right|}^{2}}d_{k-1}$$

where $\nabla g\left(U\right)={\left[\partial g/\partial u_{1},\partial g/\partial u_{2},\ldots ,\partial g/\partial u_{n}\right]}^{T}$ is the gradient vector of limit state function at point U. The iterative formula given in Equation (3) is used to compute the unit normal vector at U*_k_* based on the conjugate search direction. Figure 1 shows schematically a cycle of the conjugate search direction vector in 2D normal space. It is illustrated that $\alpha_{k+1}^{C}$ is not parallel to α*_k_*_+1_, meaning that the new point using CFORM formula is not placed on the previous points. On the other hand, the new point is tended on the previous point U*_k_*. Therefore, the CFORM may converge rapidly in comparison with FORM-based steepest descent search direction. In addition, the vector α*_k_*_+1_ is not parallel to the direction of *d*(U*_k_*) point. Therefore, stable results with no oscillations can be provided through this formulation for highly nonlinear limit state functions while the α*_k_*_+1_ and α*_k_*_−1_ may locate on a same direction in HL-RF and provide U*_k_*_+1_ = U*_k_*_−1_. This means that the HL-RF, the FSL with very large finite-step size, and CC having a large chaos control factor tended to 1 may provide unstable results for highly nonlinear problems. However, the proposed method provides stable results. Because the iterative FORM formula in Equation (2) is simply developed without any step size, the reliability index is directly computed without merit function [37], Wolfe conditions [25], sufficient descent condition [25,46], or Armijo rule [38,45]. Therefore, this method is simpler than the other modified versions of FORM formula.

### 3.2. CFORM Algorithm

The reliability index is approximated according to the proposed conjugate search direction using the following steps:

Step 1: Transforming basic random variables in *X*-space (*X* is the original random variable vector) to U-space (U is the standard normal vector) using Rosenblatt transformation as $u=\Phi^{-1}\left[F_{X}\left(x\right)\right]$ [47,48].

Step 2: Searching MPP ($U^{*}={\left(u_{1}^{*},u_{2}^{*},\ldots u_{n}^{*}\right)}^{T}$) through the use of conjugate iterative process of FORM as:

(6) $$U_{k+1}=\frac{\nabla^{T}g\left(U_{k}\right)U_{k}-g\left(U_{k}\right)}{\nabla^{T}g\left(U_{k}\right)\alpha_{k+1}^{C}}\alpha_{k+1}^{C}$$

Step 3: Calculating $\beta_{k+1}=\frac{\nabla^{T}g\left(U_{k}\right)U_{k}-g\left(U_{k}\right)}{\nabla^{T}g\left(U_{k}\right)\alpha_{k+1}^{C}}$ and checking the convergence criterion as $||U_{k}-U_{k-1}||<10^{-6}$ for the next iteration of FORM formula. The proposed iterative formula to search MPP is simple formulation and its iterative information is directly computed based on the previous results from Equation (6).

### 3.3. Validation of the Conjugate Reliability Analysis

**Figure 2:**
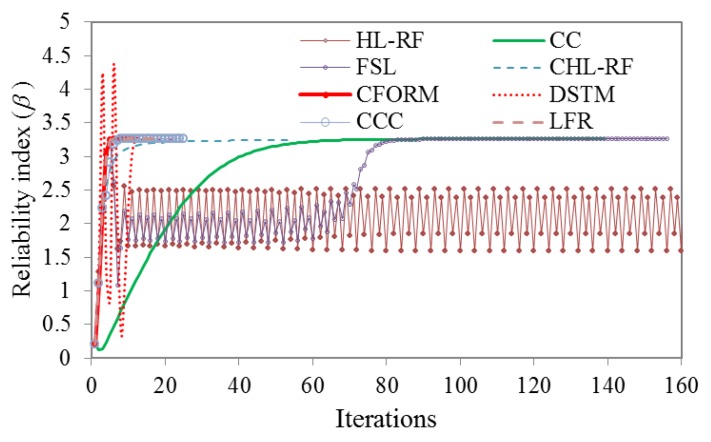
Iterative histories of HL-RF, FSL, CC, DSTM, CCC, LFR, CFORM, and CHL-RF in solving Example 1.

In this section, the performance of the developed CFORM is compared with other algorithms including HL-RF, CC, FSL, CHL-RF, DSTM, conjugate FORM-based LFR, and CCC through the use of three nonlinear limit state functions. *β* and iterations are utilized to present the efficiency and robustness of the proposed CFORM. Parameters of different reliability algorithms are set as: finite-step length (*λ*) = 50 and adjusted factor (*c*_1_) = 0.8 for FSL, *λ* = 0.1 and involutory matrix (*C*) = *I* for two algorithms of CC and DSTM, initial finite step length (*λ*_0_) = 50 and *c*_1_ = 0.8 for CHL-RF, *λ*_0_ = 50 and limited conjugate factor (*δ*) = 1 for LFR, and logistic function with parameters of initial value of 0.375 and chaos scalar factor (*a*) = 4 for CCC. Three reliability examples are considered as follows:

Example 1: A highly nonlinear reliability problem with non-normal limit state function of Equation (7) is considered [26].

(7) $$g\left(X\right)=x_{1}^{4}+x_{2}^{2}-50$$

where *x*_1_ presents the Lognormal distributed variable with the mean (*μ*) of 5 and standard deviation (*σ*) of l, and *x_2_* presents the Gumbel distribution with *μ* of 10 and *σ* of 10. The converged *β* was robustly obtained by CFORM after 11 iterations as *β* = 3.259, while *β* obtained by MCS with 1.2×10^6^ samples was 3.501. Therefore, CFORM was more robust and efficient in solving Equation (7) compared to MCS. Figure 2 shows the iterative histories of *β* obtained from HL-RF, CC, FSL, CHL-RF, and the proposed CFORM. It is shown in the figure that HL-RF method yields to 4-periodic solutions as *β* = (2.512, 1.855, 2.386, 1.597). CC and FSL robustly converged to the same *β* of 3.259, but they are less efficient than the DSTM, LFR, CCC, CHL-RF, and CFORM. Unlike the CC, DSTM, and FSL, the formulation of HL-RF is iterated without chaos feedback control factor. Therefore, as can be seen in Figure 2, the HL-RF provides periodic solutions but the improved versions of steepest descent algorithms as CC, DSTM, and FSL are robustly converged. On the other hand, the conjugate search direction in CHL-RF, CFORM, CCC, and LFR is provided by conjugate normal vector, which is not parallel to previous search directions. Therefore, as can be seen in the figure, stable results are obtained by these algorithms. The results also show the proposed method is more robust compared to HL-RF and is significantly more efficient than the other reliability methods. Notably, the CFORM converged about 5-times quicker compared with the CHL-RF method and twice quicker than CCC, DSTM, and LFR. It can also be seen in Figure 2 that the iterations of the conjugate methods of LFR, CFORM, and CCC provide same results in the initial iteration, which means that their search directions are provided using similar normalized conjugate vector. However, the convergence of CFORM is faster than the algorithms of LFR and CCC, which is because of the applied adjusting factor of $-\frac{0.1\nabla^{T}g\left(U_{k}\right)\nabla g\left(U_{k-1}\right)}{{\left||\nabla g\left(U_{k-1}\right)|\right|}^{2}}$ in the conjugate scalar factor. This factor increases the efficiency of FORM formula in the CFORM.

**Figure 3:**
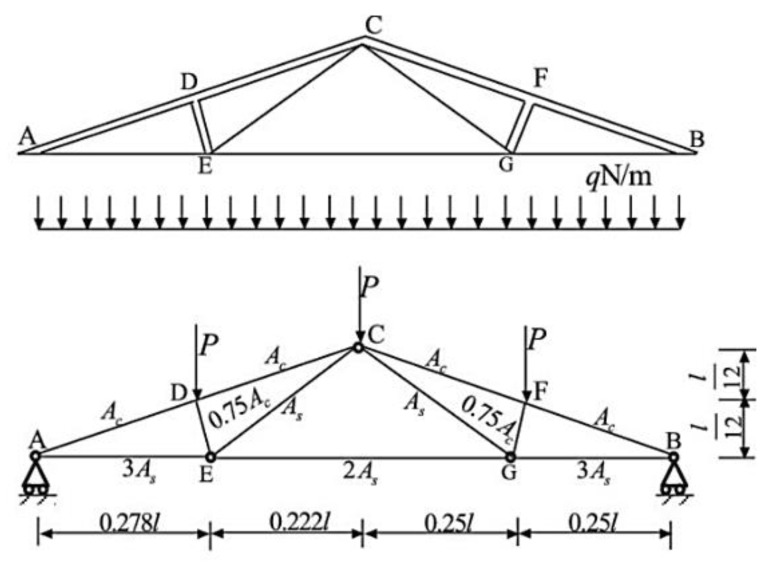
Schematic view of composite roof truss.

Example 2: A composite roof truss with compression members made of reinforced concrete, and tension bars made of steel illustrated in Figure 3 is considered with the following serviceability limit sate function:

(8) $$g=0.03-\left(\frac{ql_{}^{2}}{2}\right)\left(\frac{3.81}{AcEc}+\frac{1.13}{AsEs}\right)$$

where g is the distributed load, and *Ac*, *As*, *Ec*, *Es*, and *l* are cross-section area of reinforced concrete, cross-section area of steel bar, elastic modulus of concrete, elastic modulus of steel bar, and length of the truss member, respectively. This example included six normal independent random variables with statistical properties shown in Table 1.

**Figure 4:**
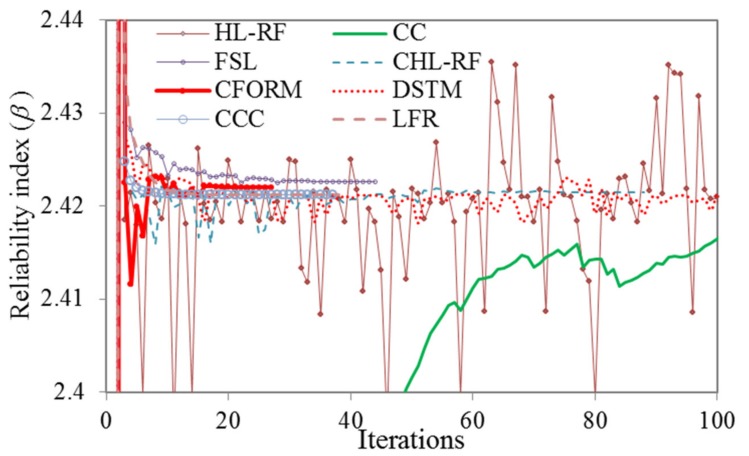
Iterative histories of HL-RF, FSL, CC, DSTM, CCC, LFR, CFORM, and CHL-RF in solving Example 2.

*β* obtained by MCS with 1.06×10^6^ samples was 2.350. The iterative histories of *β* obtained from different FORM algorithms are illustrated in Figure 4. As shown, HL-RF, DSTM, and CC algorithms have unstable results, but the CHL-RF, FSL, CCC, LFR, and proposed CFORM converged robustly to the same *β* of 2.422 after 88 (5.23 s), 44 (3.36 s), 37 (3.17 s), 38 (3.18 s), and 27 (2.61 s) iterations, respectively. The nonlinearity map of DSTM, HL-RF, and CC algorithms provides the chaotic search direction at final iterations due to their formulations while the FSL method with small finite-step length improves this drawback of the FORM-based HL-RF, CC, and DSTM. It is worth noting that, once again, the CFORM converged faster than the other converged algorithms while conjugate algorithms of CCC and LFR are shown the similar efficiency for this problem. The CFORM with nonlinear descript conjugate map provided stable results like the other conjugate approaches of CCC, LFR, and CHL-RF for this problem.

**Figure 5:**
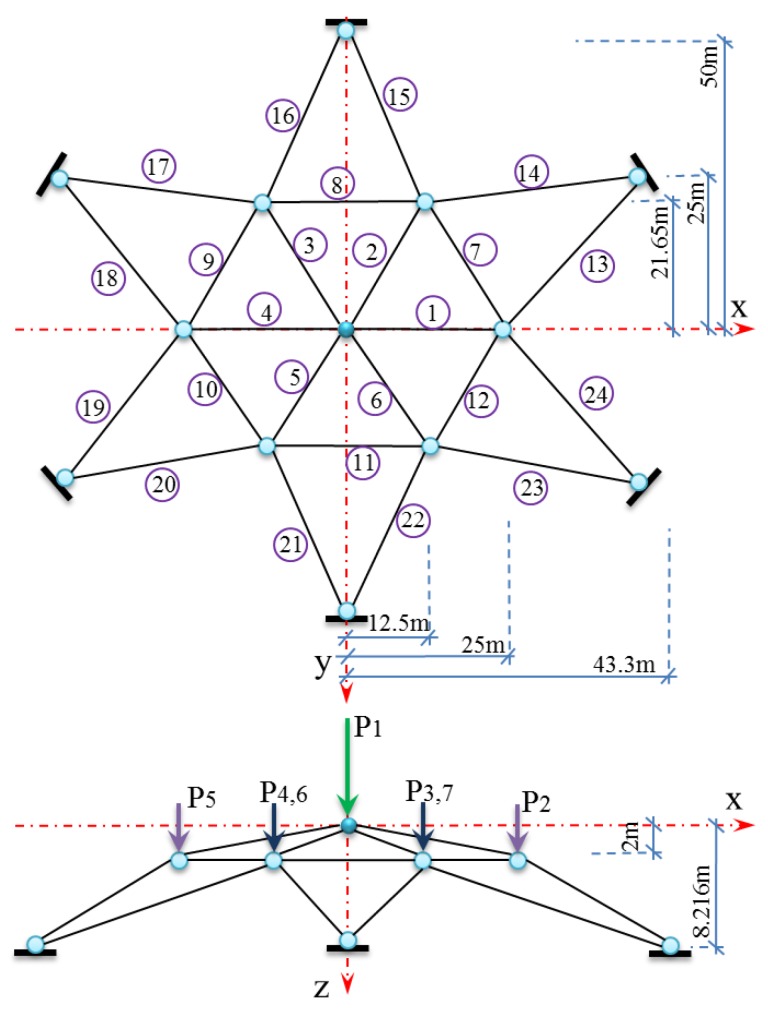
Dam truss structure for Example 3.

Example 3: A dam truss structure presented in Figure 5 is considered using the following limit state function [49]:

(9) $$g=0.01-\Delta_{}^{z}$$

where $\Delta_{}^{z}$ is the maximum displacement at z-direction. This problem involves 32 random variables as *P*_1_*–P*_7_ loads, *E* as Young’s modulus, and *A_i_* with *i*=1, 2, 3, …, 24 as cross-section of 1–24 bars components with statistical properties shown in Table 2.

**Figure 6:**
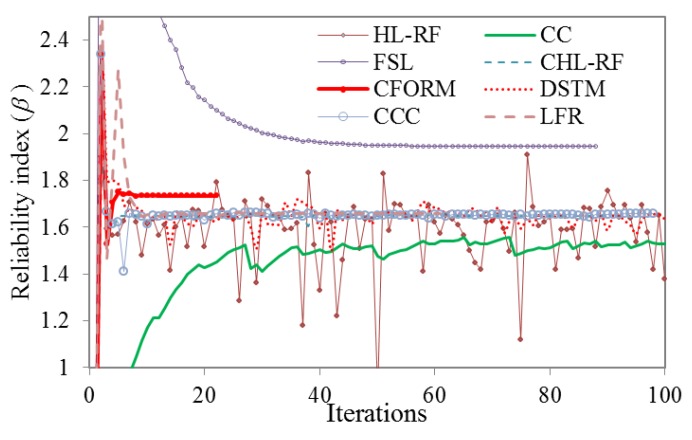
Iterative histories of HL-RF, FSL, CC, DSTM, CCC, LFR, CFORM, and CHL-RF in solving Example 3.

The converged results of *β* for different FORM algorithms are presented in Figure 6. The obtained *β* using MCS is 1.76 after 10^6^ samples with CPU-run time of 26,484 s while the proposed FORM-based CFORM and FSL algorithms are converged after 21 (49.4 s) and 88 (161.6 s) iterations to *β* of 1.735 and 1.944, respectively. As shown in Figure 6, the HL-RF, DSTM, and CC show unstable chaotic results while the conjugate methods using formulation of CCC, LFR, and CHL-RF algorithms provide stable results for reliability index of 1.657 after 91 (163.4 s), 61 (113.1 s), and 98 (166.8 s), respectively. It can be conducted that the conjugate scalar factor combined with the previous conjugate vector may improve the accuracy of the results of this problem in comparison with the other FORM-based conjugate search direction as CCC, LFR, and CHL-RF while proposed CFORM closely agrees with the results of MCS compared to the FSL. Therefore, the CFORM is more robust in comparison with the FORM-based HL-RF, CC, and DSTM and it is significantly more accurate and efficient in comparison with the CCC, LFR, and CHL-RF.

The results of the three examples indicate that HL-RF, CC, and DSTM algorithms provide unstable solutions, whereas the CFORM, CCC, LFR, and FSL robustly converge. The CFORM converges quicker compared to the other reliability algorithms-based conjugate search direction of LFR, CCC, and CHL-RF. These observations indicate that the proposed CFORM provided superior results compared to existing reliability methods in terms of efficiency and robustness; hence it is selected in this study for the reliability analysis of FRP-confined concrete.

## 4. Probabilistic Modeling of FRP-Confined Concrete

For reliability analysis of FRP-confined concrete, two major limit state functions can be defined based on *f′_cc_* and *ε_cu_* of FRP-confined concrete as follows:

(10) $$g\left(f\right)=\kappa_{f}{\left(f_{cc}^{\prime }/f_{co}^{\prime }\right)}^{Mod}-1$$

(11) $$g\left(\varepsilon \right)=\kappa_{\varepsilon }{\left(\varepsilon_{cu}/\varepsilon_{co}\right)}^{Mod}-1.5$$

where *f′_cc_*, *f′_co_*, *ε_cu_*, and *ε_co_* denote ultimate compressive strength, unconfined concrete strength, ultimate axial strain, and axial strain corresponding to *f′_co_*, respectively. (*f′_cc_*/*f′_co_*)*^Mod^* and (*ε_cu_*/*ε_co_*)*^Mod^* represent the strength and strain enhancement ratio defined using mathematical models, respectively. *κ_f_* and $\kappa_{\varepsilon }$ are the model error for *f′_cc_* and *ε_cu_* of FRP-confined concrete models, respectively. To express the uncertainties between the prediction and experimental ultimate condition of FRP-confined concrete, *κ_f_* and $\kappa_{\varepsilon }$ are used as the ratio of the experimental data to the predicted points of strength and strain enhancement ratio, respectively, as follows:

(12) $$\kappa_{f}=\frac{{\left(f_{cc}^{\prime }/f_{co}^{\prime }\right)}^{exp}}{{\left(f_{cc}^{\prime }/f_{co}^{\prime }\right)}^{Mod}}$$

(13) $$\kappa_{\varepsilon }=\frac{{\left(\varepsilon_{cu}/\varepsilon_{co}\right)}^{exp}}{{\left(\varepsilon_{cu}/\varepsilon_{co}\right)}^{Mod}}$$

The models proposed by Ozbakkaloglu and Lim [50] (Equations (14) and (15)) are used to approximate the strength and strain enhancement ratio in the probabilistic modeling.

(14) $${\left(\frac{f_{cc}^{\prime }}{f_{co}^{\prime }}\right)}^{Mod}=1+0.0058\frac{K_{l}}{f_{co}^{\prime }}+3.22\left(\frac{f_{lu,a}-f_{lo}}{f_{co}^{\prime }}\right)$$

(15) $${\left(\frac{\varepsilon_{cu}}{\varepsilon_{co}}\right)}^{Mod}=2-\frac{f_{co}^{\prime }-20}{100}+0.271{\left(\frac{K_{l}}{f_{co}^{\prime }}\right)}^{0.9}\frac{\varepsilon_{h,rup}^{1.35}}{\varepsilon_{co}}$$

where $K_{l}$, $f_{lo}$, and $f_{lu,a}$ are confinement stiffness of the FRP shell, threshold confining pressure, and actual confining pressure defined as follows, respectively:

(16) $$K_{l}=\frac{2E_{f}t_{f}}{D}$$

(17) $$f_{lo}=K_{l}\left(0.43+0.009\frac{K_{l}}{f_{co}^{\prime }}\right)\varepsilon_{co}$$

(18) $$f_{lu,a}=K_{l}\varepsilon_{h,rup}=K_{l}\left(0.9-2.3f_{co}^{\prime }\times 10^{-3}-0.75E_{f}\times 10^{-6}\right)\varepsilon_{f}$$

where *ε_h,rup_* is the FRP hoop rupture strain; *ε_f_* is the ultimate tensile strength, *t_f_* is the total thickness, and *E_f_* is the elastic modulus of fibers used in FRP jackets; and *D* is the diameter of specimens. *ε_co_* is determined using Equation (19) [51].

(19) $$\varepsilon_{co}=\frac{f_{co}^{\prime \;0.225}}{1000}{\left(\frac{152}{D}\right)}^{0.1}{\left(\frac{2D}{H}\right)}^{0.13}$$

where *H* is the specimen height in millimeter.

**Figure 7:**
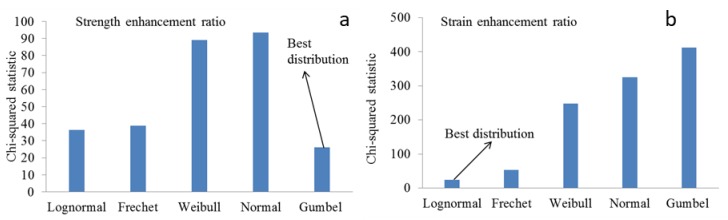
Chi-squared statistics of different distribution functions for model errors in the prediction of (**a**) strength and (**b**) strain enhancement ratio.

The statistical indicators, such as mean, coefficients of variation, and probability distribution function (PDF) are calculated to determine *κ_f_* and *κ_ε_*. The probability distribution parameters of Frechet, Gumbel, Weibull, Lognormal, and Normal distribution functions for the model errors are estimated by the maximum likelihood method and the best distribution function is selected using the chi-squared statistic (see Reference [38] for details). Figure 7 shows chi-squared statistic of different distributions for model errors of strength and strain enhancement ratios. As can be seen in the figure, the Gumbel and Lognormal distributions are the best fitting distribution for strength and strain enhancement ratios, respectively.

**Figure 8:**
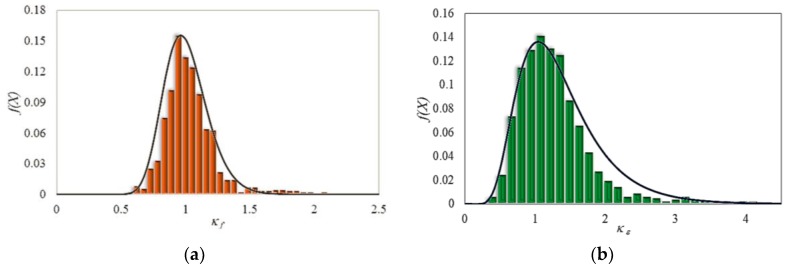
Histogram and best probability density functions for model errors of (**a**) strength (*κ_f_*) and (**b**) strain (*κ_ε_*) enhancement ratio.

The histogram of data, Gumbel distribution function for model error of strength enhancement ratio and Lognormal distribution function for model error of strain enhancement ratio are illustrated in Figure 8. As observed, *μ* of 1.005 and coefficients of variation (COV) of 0.184 were obtained for the model uncertainty of strength enhancement ratio and *μ* of 1.195 and COV of 0.247 were obtained for strain enhancement ratio.

To investigate the effect of FRP material properties (i.e., *E_f_* and *t_f_*) on the probabilistic characteristics of the model error, six categories are considered for FRP confinement based on the fiber type and *E_f_*, including three categories for specimens confined by carbon FRP (CFRP), two categories for glass FRP (GFRP), and one category for aramid FRP (AFRP). A large experimental database containing 769 cylindrical concrete specimens confined by unidirectional fibers in hoop direction was used in the probabilistic analysis. A total of 607 data points were compiled from References [14] and [50], with the additional 162 data points from more recently published studies [52,53,54,55]. The database includes NSC (i.e., *f′_co_* < 50 MPa) and HSC (i.e., *f′_co_* ≥ 50 MPa) specimens confined by i) low (i.e., *E_f_* ≤ 190 GPa), normal (i.e., 190 < *E_f_* ≤ 260 GPa), and high (i.e., *E_f_* > 260 GPa) modulus carbon fibers, ii) lower (i.e., *E_f_* ≤ 60 GPa) and higher modulus (i.e., *E_f_* > 60 GPa) glass fibers, and iii) aramid fibers under light (actual confinement ratio (*f_lu,a_*/*f’_co_*) ≤ 0.2), moderate (0.2 < *f_lu,a_*/*f’_co_* ≤ 0.5), and heavy (*f_lu,a_*/*f’_co_* > 0.5) confinement levels.

The FRP material properties of the specimens in the previously mentioned six database segments are presented in Table 3.

The Normal, Lognormal, Frechet, Gumbel, and Weibull distributions are used to determine the best PDF in evaluating the properties of FRP-confined concrete. The parameters of PDF are evaluated by the maximum likelihood estimator. The results of statistical analysis for each of the previously defined specimen categories are illustrated in Table 4. These are used for reliability analysis of database of FRP-confined concrete columns. As observed, the best probability distributions for $\kappa_{f}$ and *κ_ε_* are given by the Normal, Lognormal, Gumbel, and Weibull distributions. In addition, Gumbel, Normal, and Lognormal distributions provided the best fitness on *κ_ε_* and $\kappa_{f}.$ The COV values of $\kappa_{f}$, *κ_ε_*, and *ε_f_* varied from 0.103 to 0.175, 0.168 to 0.382, and 0.218 to 0.391, respectively. Conversely, only a small variation was observed in the COV (i.e., 0.076–0.113 and 0.074–0.125) of other FRP properties (i.e., *t_f_* and *E_f_*, respectively). Therefore, it is suggested that COV of *t_f_* and *E_f_* can be taken to be 0.08 and 0.1, respectively. As can be seen in Table 4, Weibull or Gumbel distribution can be used for *t_f_*, whereas Gumbel or Lognormal distribution is better fitted for *E_f_* which is denoted to results extracted from Reference [39]. It is also assumed that *f′_co_*, *H*, and *D* follow the Normal distribution with COV of 0.18, 0.1, and 0.1, respectively [56]. The selection of the Normal distribution was based on the fact that this distribution is symmetric with respect to its mean and hence has a same effect on all test results in the database [56,57].

## 5. Reliability Analysis of FRP-Confined Concrete

The reliability analysis is implemented on the experimental data points of six FRP categories. Based on the results from the probabilistic modeling, the reliability index is determined using the proposed CFORM method. Therefore, a probabilistic model is developed based on the reliability analysis of *f′_cc_* and *ε_cu_* of FRP-confined concrete and then *β* is determined according to the probabilistic model for each FRP category.

### 5.1. Limit State Function for f′_cc_ and ε_cu_ Results in the Database

Various uncertainties in this type of structural reliability analysis are given using the probabilistic models. Generally, the safety level-based reliability analyses of FRP-confined concrete database can be estimated based on a mathematical relation, in which the safe domain is separated into failure domain with respect to various uncertainties using a probabilistic model. The reliability index is then determined based on the limit state functions presented in Equations (20) and (21) which are determined by rearranging empirical models in Equations (14) and (15) into Equations (10) and (11) for evaluation of the safety level of FRP-confined concrete database.

(20) $$g\left(f\right)=\kappa_{f}f_{co}^{\prime }\left[1+0.0058\frac{K_{l}}{f_{co}^{\prime }}+3.22\left(\frac{f_{lu,a}-f_{lo}}{f_{co}^{\prime }}\right)\right]-f_{co}^{\prime }$$

(21) $$g\left(\varepsilon \right)=\kappa_{\varepsilon }\varepsilon_{co}[2-\frac{f_{co}^{\prime }-20}{100}+0.271\left(\frac{K_{l}}{f_{co}^{\prime }}{)}^{0.9}\frac{\varepsilon_{h,rup}^{1.35}}{\varepsilon_{co}}\right]-1.5\varepsilon_{co}$$

in which g < 0 and g > 0 denote failure and safe domains for *f′_cc_* and *ε_cu_*, respectively. Based on the limit state functions, the reliability index of concrete confined by FRP is evaluated by eight random variables of *f′_co_*, *D*, *H*, *t_f_*, *E_f_*, *ε_f_*, *κ_f_*, and *κ_ε_*. The means, COVs, and PDFs for the two variables of *κ_f_* and *κ_ε_* are given based on the presented results in Table 4 while the means of the other random variables are given based on the values of data points.

### 5.2. Reliability Analysis of the Database

**Figure 9:**
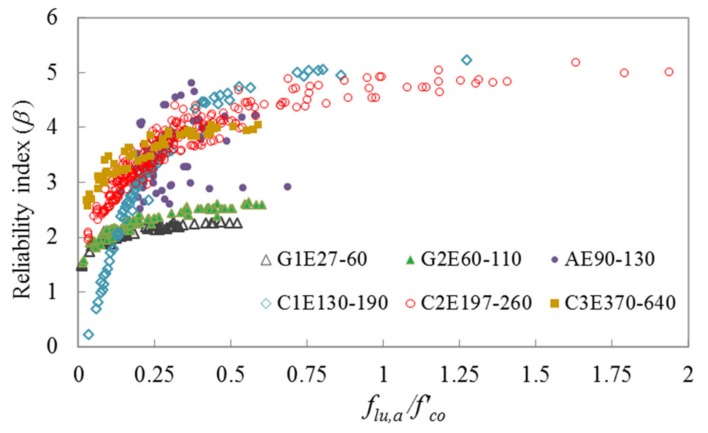
Variation of *β* with *f_lu,a_*/*f’_co_* for different segments of the FRP-confined concrete test database using limit state function of strength capacity.

**Figure 10:**
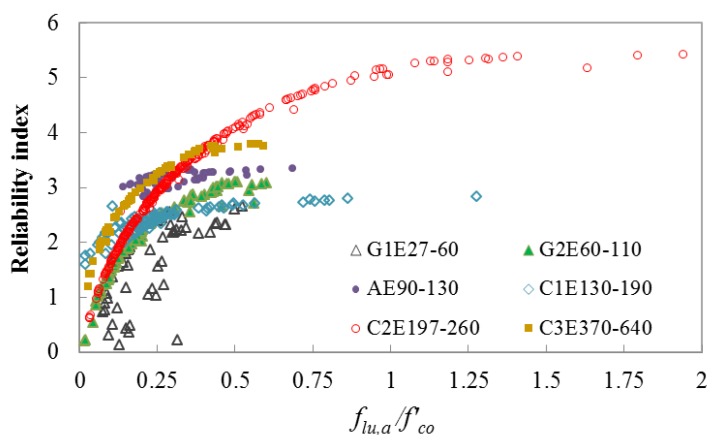
Variation of *β* with *f_lu,a_*/*f’_co_* for different segments of the FRP-confined concrete test database using limit state function of strain capacity.

For assessing the safety levels of different segments of the current database of FRP-confined concrete, *β* is computed using the CFORM algorithm for different FRP categories based on the limit state functions given in Equations (20) and (21). Figure 9 and Figure 10 show the relationships between *β* and *f_lu,a_*/*f’_co_* for different FRP categories, which are respectively computed using limit state functions of g(f) and g(ε). The strength and strain capacity of the specimens are improved for *f_lu,a_*/*f’_co_* of more than 1, owing to the translation of hoop stress between the concrete core and FRP sheet.

Four safety levels are defined for the segments of the database based on the reliability analysis results, namely: low (*β* < 2.5), moderate (2.5 ≤ *β* ≤ 3.25), high (3.25 < *β* ≤ 4.5), and very high (4.5 < *β*) levels. Table 5 shows the influence domains of low to very high safety levels for different categories.

As shown in Table 5, the database containing specimens confined by GFRP, except G2E60-110 group with 30 MPa ≤ *f’_co_* ≤ 50 MPa and *f_lu,a_*/*f’_co_* > 0.3, and CFRP with 35 MPa ≤ *f’_co_* ≤ 170 MPa and *f_lu,a_*/*f’_co_* ≤ 0.15 falls under the low safety level. The part of the database containing the specimens confined by GFRP with 60 GPa ≤ *E_f_* ≤ 110 GPa, *f’_co_* ≤ 50 MPa, and 0.31 ≤ *f_lu,a_*/*f’_co_* ≤ 0.71; CFRP with *E_f_* ≤ 260 GPa, 35 MPa ≤ *f’_co_* ≤ 170 MPa, and 0.15 < *f_lu,a_*/*f’_co_* ≤ 0.31, and *E_f_* ≥ 370 GPa, 30 MPa ≤ *f’_co_* ≤ 85 MPa, and 0.14 < *f_lu,a_*/*f’_co_* ≤ 0.25; and AFRP with 40 MPa ≤ *f’_co_* ≤ 120 MPa and 0.14 ≤ *f_lu,a_*/*f’_co_* ≤ 0.3 exhibits a moderate safety level. The section of the database containing the specimens confined by CFRP with *E_f_* ≤ 260 GPa, 30 MPa ≤ *f’_co_* ≤ 110 MPa, and 0.31 < *f_lu,a_*/*f’_co_* ≤ 0.7, and *E_f_* ≥ 370 GPa, *f’_co_* ≤ 40 MPa, and 0.25 < *f_lu,a_*/*f’_co_* ≤ 0.75; and AFRP with 35 MPa ≤ *f’_co_* ≤ 110 MPa and 0.3 < *f_lu,a_*/*f’_co_* ≤ 0.64 exhibits a high safety level, and that containing NSC specimens confined by CFRP with *E_f_* ≤ 260 GPa, 20 MPa ≤ *f’_co_* ≤ 35 MPa, and 0.7 < *f_lu,a_*/*f’_co_* ≤ 2.13; and AFRP with *f’_co_* ≤ 30 MPa and 0.3 ≤ *f_lu,a_*/*f’_co_* ≤ 0.5 exhibits a very high safety level.

Based on the results presented in Table 5 it is recommended that the segments of the database with a high and very high safety level can be used confidently in the future for model development and validation of FRP-confined concrete. However, the parts of the database with a low and moderate safety level require additional experimental results before they can be reliably used for design and modeling purposes. The proposed CFORM provided robust and efficient results for evaluating the reliability of the existing test database of the ultimate condition of FRP-confined concrete (*f′_cc_* and *ε_cu_*). However, the accuracy of the proposed probabilistic framework is dependent on the accuracy of the predictions of the performance function. Therefore, it is recommended that the future experimental studies on FRP-confined concrete target segments of the database with a low and moderate safety level to propose an accurate empirical model (performance function) for predicting the ultimate condition of FRP-confined concrete columns.

## 6. Conclusions

In this paper, a probabilistic model was developed to evaluate the failure probability of *f′_cc_* and *ε_cu_* of concrete confined by FRP. *f′_cc_* and *ε_cu_* of FRP-confined concrete were determined based on a FORM with conjugate search direction, which is called CFORM. Three nonlinear reliability problems were utilized to validate the efficiency and accuracy of the CFORM compared with existing reliability methods. Reliability analysis results demonstrated that the CFORM is more robust and efficient in comparison with the existing methods. CFORM was then implemented for evaluating the safety levels of *f′_cc_* and *ε_cu_* results in the current database of FRP-confined concrete. Four safety levels were defined for six categories based on *f’_co_* and *f_lu,a_*/*f’_co_*. The following conclusions are drawn from the reliability analysis of FRP-confined concrete:

(1) Lognormal and Gumbel distributions provide the best fitness for *κ_f_* and *κ_ε_* of FRP-confined concrete.
(2) The segments of the database containing (i) NSC heavily confined (i.e., *f_lu,a_*/*f’_co_* > 0.5) by low and normal modulus carbon fibers (i.e., *E_f_* ≤ 260 GPa), and (ii) NSC moderately confined (i.e., 0.3 ≤ *f_lu,a_*/*f’_co_* ≤ 0.5) by aramid fibers represent a very high safety level.
(3) The segments of the database containing moderately and heavily confined (i.e., *f_lu,a_*/*f’_co_* > 0.2) (i) NSC by high modulus carbon fibers (i.e,. *E_f_* > 260 GPa), (ii) HSC by low and normal modulus carbon fibers (i.e., *E_f_* ≤ 260 GPa), and iii) HSC by aramid fibers exhibit a high safety level.
(4) The segments of the database containing (i) NSC moderately and heavily confined (i.e., *f_lu,a_*/*f’_co_* > 0.3) by higher modulus glass fibers (i.e., *E_f_* > 60 GPa), (ii) HSC lightly confined (i.e., *f_lu,a_*/*f’_co_* ≤ 0.2) by highly modulus carbon fibers (i.e., *E_f_* > 260 GPa), and (iii) HSC lightly confined (i.e., *f_lu,a_*/*f’_co_* ≤ 0.2) by aramid fibers exhibit a moderate safety level.
(5) The segments containing (i) HSC lightly and moderately confined (i.e., *f_lu,a_*/*f’_co_* ≤ 0.5) by glass fibers, and (ii) HSC lightly confined (i.e., *f_lu,a_*/*f’_co_* ≤ 0.15) by low and normal modulus carbon fibers (i.e., *E_f_* ≤ 260 GPa) represent a low safety level.

Additional experimental studies targeting the segments of the database with a low and moderate safety level are recommended to improve these parts of the database, so that it can be used reliably for design and modeling purposes. It is also suggested to use the proposed CFORM in fuzzy reliability analysis to prevent any uncertainties in the statistical properties of FRP-confined concrete that may arise from the quality of the preparation process of the specimen, measurement error, and inherent uncertainties.

## Figures and Tables

**Table 1:** Random variables for roof truss.

Random Variable	g	*l* (m)	*A*s (*m*^2^)	*A*c (m^2^)	*E*s (Pa)	*E*c (Pa)
Mean	20,000	12	9.82 × 10^−4^	0.04	1 × 10^11^	2 × 10^10^
Standard deviation	1400	0.12	5.98 × 10^−5^	0.0048	6 × 10^9^	1.2 × 10^9^

**Table 2:** Random variables for dam truss example.

Random Variable	*A*_1_–*A*_6_ (m^2^)	*A*_7_–*A*_12_ (m^2^)	*A*_13_–*A*_24_ (m^2^)	*E* (GPa)	*P*_1_ (kN)	*P*_2_–*P*_7_ (kN)
Mean	0.013	0.01	0.016	205	20	10
COV	0.1	0.1	0.1	0.12	0.15	0.12
Distribution	Normal	Normal	Normal	Normal	Gumbel	Gumbel

**Table 3:** Fiber material properties for different segments of the fiber-reinforced polymer (FRP)-confined concrete database.

Parameter	C1E130-190	C2E197-260	C3E370-640	G1E27-60	G2E60-110	AE90-130
*E_f_* (GPa)	130–190	197–260	370–640	27–60	60–110	90–130
*ε_f_* (%)	0.67–1.52	1.50–2.65	0.41–1.20	2.00–3.38	2.11–4.30	1.74–3.96
*t_f_* (*mm*)	0.10–3.51	0.20–2.26	0.15–0.85	0.60–3.90	0.15–2.55	0.15–1.20

**Table 4:** Statistics of model error and fiber material properties of different segments of the current database of FRP-confined concrete.

Categories	Data	Variable	Mean	COV	PDF
C1E130-190	93	*t_f_* (*mm*)	1.198	0.085	Lognormal
		*ε_f_* (%)	1.01	0.352	Gumbel
		*E_f_* (GPa)	154.5	0.125	Lognormal
		*κ_ε_*	1.207	0.337	Gumbel
		*κ* *_f_*	0.929	0.133	Gumbel
C2E197-260	284	*t_f_* (*mm*)	1.124	0.081	Weibull
		*ε_f_* (%)	1.95	0.291	Gumbel
		*E_f_* (GPa)	237.5	0.086	Lognormal
		*κ_ε_*	1.068	0.382	Lognormal
		*κ* *_f_*	0.997	0.175	Lognormal
C3E370-640	61	*t_f_* (*mm*)	0.385	0.089	Gumbel
		*ε_f_* (%)	0.77	0.327	Lognormal
		*E_f_* (GPa)	418.6	0.095	Frechet
		*κ_ε_*	1.095	0.220	Lognormal
		*κ* *_f_*	1.039	0.103	Weibull
G1E27-60	58	*t_f_* (*mm*)	2.262	0.076	Weibull
		*ε_f_* (%)	2.57	0.377	Lognormal
		*E_f_* (GPa)	34.7	0.074	Lognormal
		*κ_ε_*	1.098	0.418	Lognormal
		*κ* *_f_*	1.007	0.165	Gumbel
G2E60-110	82	*t_f_* (*mm*)	0.921	0.113	Gumbel
		*ε_f_* (%)	3.12	0.391	Gumbel
		*E_f_* (GPa)	82.2	0.116	Lognormal
		*κ_ε_*	1.142	0.362	Lognormal
		*κ* *_f_*	0.97	0.161	Gumbel
AE90-130	63	*t_f_* (*mm*)	0.751	0.095	Weibull
		*ε_f_* (%)	2.62	0.218	Gumbel
		*E_f_* (GPa)	120.1	0.085	Gumbel
		*κ_ε_*	1.284	0.167	Lognormal
		*κ* *_f_*	0.909	0.116	Lognormal

**Table 5:** Confinement ratio and unconfined concrete strength ranges for different safety levels of the current database of FRP-confined concrete using two ultimate failure modes.

Safety Level		C1E130-190		C2E197-260		C3E370-640		G1E27-60		G2E60-110		AE90-130	
		*f_lu,a_*/*f’_co_*	*f’_co_* (MPa)	*f_lu,a_*/*f’_co_*	*f’_co_* (MPa)	*f_lu,a_*/*f’_co_*	*f’_co_* (MPa)	*f_lu,a_*/*f’_co_*	*f’_co_* (MPa)	*f_lu,a_*/*f’_co_*	*f’_co_* (MPa)	*f_lu,a_*/*f’_co_*	*f’_co_* (MPa)
Low	*β* < 2.5	0.03–0.15	35–170	0.07–0.15	50–130	N.A. *	N.A.	0.01–0.50	25–110	0.01–0.30	35–110	N.A.	N.A.
Moderate	2.5 ≤ *β* ≤ 3.25	0.16–0.20	30–130	0.16–0.31	35–170	0.14–0.25	30–85	N.A.	N.A.	0.31–0.71	30–50	0.14–0.3	40–120
High	3.25 < *β* ≤ 4.5	0.21–0.5	30–130	0.32–0.70	30–110	0.26–0.75	25–40	N.A.	N.A.	N.A.	N.A.	0.31–0.64	35–110
Very high	*β* > 4.5	0.51–1.28	25–45	0.71–2.13	20–35	N.A.	N.A.	N.A.	N.A.	N.A.	N.A.	0.30–0.50	25–30

* N.A.: Not applicable, which indicates that it was not possible to assess that segment of the database because of the lack of availability of test results.

## Nomenclature

AFRP	Aramid fiber-reinforced polymer
CC	Chaos control
CCC	Chaotic conjugate control
CFORM	Conjugate first-order reliability method
CFRP	Carbon fiber-reinforced polymer
CHL-RF	Conjugate Hasofer-Lind and Rackwitz-Fiessler method
COV	Coefficients of variation
DSTM	Directional stability transformation method
FORM	First-order reliability method
FR	Fletcher and Reeves
FRP	Fiber-reinforced polymer
FSL	Finite-step length method
GFRP	Glass fiber-reinforced polymer
HL-RF	Hasofer-Lind and Rackwitz-Fiessler method
HSC	High strength concrete
IHL-RF	Improved Hasofer-Lind and Rackwitz-Fiessler method
LFR	Limited Fletcher and Reeves
MCS	Monte Carlo simulation
MPP	Most probable point
NSC	Normal strength concrete
PDF	Probability distribution function
SORM	Second-order reliability method
STM	Stability transformation method
*a*	Chaos scalar factor
*g*	Distributed load
*l*	Length of truss
*C*	Involutory matrix
*D*	Diameter of concrete specimen
*E*	Young’s modulus
*H*	Height of concrete specimen
*P*	Concentric load
U	Normal standard random variables vector
*X*	Original random variable vector
*As*	Cross sectional areas of steel bars
*c*_1_	Adjusted factor
*d_k_*	Conjugate search direction vector
*d*(U*_k_*)	Point-based conjugate search direction
*Ec*	Elastic modulus of reinforced concrete
*E_f_*	Elastic modulus of fibers used in FRP jacket
*Es*	Elastic modulus of steel bars
$f_{lo}$	Threshold confining pressure
*f_lu,a_*	Actual confining pressure
*f_X_*	Joint probability density function
*g(X)*	Limit state function
*g(X)* ≤ 0	Failure region
$K_{l}$	Confinement stiffness of the FRP shell
*P_f_*	Failure probability
*t_f_*	Total thickness of fibers used in FRP jacket
*f′_cc_*	Concrete ultimate compressive strength
*f′_co_*	Unconfined concrete strength
*U**	Most probable point (MPP) in *U*-space
α_*k*+1_	Unit normal vector
$\alpha_{k+1}^{C}$	Conjugate unit normal vector at design point U*_k_*
*β*	Reliability index
*λ*	Finite-step length
*δ*	Limited conjugate factor
*σ*	Standard deviation
*μ*	Mean
*ε_co_*	Axial strain corresponding to the *f′_co_*
*ε_cu_*	Ultimate axial strain
*ε_f_*	Ultimate tensile strength of fibers used in FRP jacket
*ε_h,rup_*	Hoop rupture strain of the FRP
*κ_f_*	Model error for ultimate strength
$\kappa_{\varepsilon }$	Model error for ultimate strain
Φ	Standard normal cumulative distribution function
$\Delta_{}^{z}$	Maximum displacement at z-direction
∇g(U)	Gradient vector of the limit state function at point U
